# Fronto-temporal brain systems supporting spoken language comprehension

**DOI:** 10.1098/rstb.2007.2158

**Published:** 2007-09-07

**Authors:** Lorraine K Tyler, William Marslen-Wilson

**Affiliations:** 1 Centre for Speech, Language and the Brain, Department of Experimental Psychology, University of CambridgeDowning Street, Cambridge CB1 3EB, UK; 2 MRC Cognition and Brain Sciences Unit15 Chaucer Road, Cambridge CB2 2EF, UK

**Keywords:** fronto-temporal language system, connectivity analysis, neural language system

## Abstract

The research described here combines psycholinguistically well-motivated questions about different aspects of human language comprehension with behavioural and neuroimaging studies of normal performance, incorporating both subtractive analysis techniques and functional connectivity methods, and applying these tasks and techniques to the analysis of the functional and neural properties of brain-damaged patients with selective linguistic deficits in the relevant domains. The results of these investigations point to a set of partially dissociable sub-systems supporting three major aspects of spoken language comprehension, involving regular inflectional morphology, sentence-level syntactic analysis and sentence-level semantic interpretation. Differential patterns of fronto-temporal connectivity for these three domains confirm that the core aspects of language processing are carried out in a fronto-temporo-parietal language system which is modulated in different ways as a function of different linguistic processing requirements. No one region or sub-region holds the key to a specific language function; each requires the coordination of activity within a number of different regions. Functional connectivity analysis plays the critical role of indicating the regions which directly participate in a given sub-process, by virtue of their joint time-dependent activity. By revealing these codependencies, connectivity analysis sharpens the pattern of structure–function relations underlying specific aspects of language performance.

## 1. Introduction

In understanding normal spoken language, the listener is confronted with a flow of rapidly accumulating and dynamically varying acoustic–phonetic information. This needs to be broken down into its constituent words and morphemes so that the information carried by these primary linguistic units can be used to construct an interpretation of the message being transmitted, weaving together cues to the linguistic structure and the meaning. Over the last decade, a framework has begun to emerge for understanding these capacities from a cognitive neuroscience perspective. This cross-disciplinary perspective combines novel inputs from the neurobiology of primate auditory processing systems and from structural and functional neuroimaging of the intact and damaged human brain, with the older traditions of the neurological and neuropsychological study of language and the brain.

An outcome of this combination of sources is a renewed emphasis on the bi-hemispheric foundations of primate auditory and human speech communication systems, moving away from the classical view of language as a purely left hemisphere (LH) phenomenon, as exemplified in the standard Broca–Wernicke–Lichtheim diagram (figure 1). Recent research with non-human primates highlights the underlying hemispheric symmetry of the auditory processing systems upon which human speech comprehension systems are presumably built, although several aspects of critically linguistic (as opposed to auditory) processing show clear asymmetries, as we describe below. These studies with non-human primates show that bilateral inputs to auditory processing areas in superior temporal cortex (core, belt and parabelt) link to major processing streams, running dorsally and ventrally to processing regions in frontal cortex, inferior parietal areas and other temporal lobe areas (e.g. Kaas & Hackett 1999; Rauschecker & Tian 2000).

Although this analysis has increasingly been adopted as a template for thinking about the organization of the speech and language processing system in the human brain (e.g. Hickok & Poeppel 2000; Scott & Johnsrude 2003), two major caveats are in order. The first is that the human brain diverges in many respects from the macaque brain, but most extensively in the anterior temporal lobe and frontal lobe areas that are critically involved in the systems postulated—for example, the macaque entirely lacks the middle temporal gyrus that is a prominent and functionally significant part of the human brain. The second is that a system designed to support spoken language will need to make different and additional functional demands to those served by the macaque system. Nonetheless, the emergence of a well-specified account of the neurobiological underpinnings of primate auditory processing has had important consequences. It provides a model for what a theory of these systems needs to look like, in terms of the specificity of both the functional and the neural account that is provided, and it suggests a very different approach to the characterization of human language function.

Classical cognitive and psycholinguistic approaches to the functional structure of the system for mapping from sound to meaning have always assumed that a single, unitary process (or succession of processes) is engaged to carry out this mapping. This is reflected in the focus in these models on a single neural system involving inferior frontal cortex (especially Broca's area) and posterior temporal cortex (Wernicke's area) and the major white matter tract (the arcuate fasciculus) that connects them (figure 1). The neurobiological evidence suggests, however, that the underlying neural system is not organized along these lines and that multiple parallel processing streams are involved, extending hierarchically outwards from auditory cortex in both posterior and anterior directions. Instead of language being processed primarily within a single dorsal stream, strong evidence is emerging that a substantial ventral stream is also involved (Hickok & Poeppel 2004). This is thought to extend from posterior temporo-parietal sites via the superior and middle temporal cortex to the anterior temporal and orbito-frontal cortex, by means of the white matter tracts of the inferior longitudinal fasciculus and the uncinate fasciculus. Although these dorsal and ventral streams potentially exist in both hemispheres, recent research suggests that there are considerable hemispheric asymmetries. For example, using new techniques such as diffusion tensor imaging (DTI), which examine the structure of white matter tracts, Buchel *et al*. (2004) have shown an increased white matter composition of the left arcuate fasciculus and inferior longitudinal fasciculus in healthy subjects. Other studies using DTI tractography have suggested that there may be even more marked hemispheric asymmetry. Parker *et al*. (2005), for example, have claimed that while the arcuate fasciculus is reliably observed in both the hemispheres across subjects, the ventral stream is only seen in the LH.

This anatomical asymmetry seems to be reflected in functional asymmetry. Although a combination of inputs from both novel and historical sources lend some support to both the active role of right hemisphere (RH) structures in language function and the existence of multiple processing streams (although these tend to be more left lateralized), the role of the RH appears to be limited. A number of functional imaging studies have shown that speech processing in humans activates bilateral temporal lobe structures in and around primary auditory processing areas (e.g. Zatorre *et al*. 1992; Zatorre & Gandour 2008; though see Scott & Wise (2003) for a critique of some earlier studies). Moreover, this bilateral activation is not only limited to low-level acoustic and phonetic analyses, but also implicates lexical analysis processes—mapping the speech input onto lexical representations (Binder *et al*. 2000; Scott & Wise 2004), as reflected both in studies using haemodynamic techniques such as PET and fMRI and those using electrophysiological techniques such as EEG and MEG (Marinkovic *et al*. 2003).

RH involvement at these levels is consistent with the neuropsychological evidence that patients with extensive damage to LH perisylvian language areas (L frontal and superior temporal regions) but spared RH can still recognize spoken words and access lexical meaning (Tyler *et al*. 1995*a*, 2002*a*; Dronkers *et al*. 2004). For example, such patients typically show semantic priming effects and reaction times within the normal range for spoken words—but only when they are morphologically simple, such as *desk, rabbit*, etc. (Tyler *et al*. 1995*a*,*b*, 2002*a*,*b*; Longworth *et al*. 2005). These normal priming effects suggest that the patients do not access semantic information more slowly than unimpaired listeners, contrary to earlier claims that patients with left perisylvian lesions are slow to access semantic information (Milberg *et al*. 1987). Moreover, given that the patients produce normal patterns of priming in the face of extensive LH damage, this suggests that the RH can support quite extensive processing of simple words.

Nonetheless, despite this evidence for a degree of bilateral parallelism in some aspects of language function, it is also clear that the most critical language functions depend on an intact left-dominant perisylvian core language system, linking left inferior frontal cortex (LIFC) with temporal and posterior parietal cortices. Damage to these regions can cause a permanent disruption of some key language functions while damage to parallel regions on the right generally does not. A particularly salient feature here is the disruption of the combinatorial aspects of language function—those processes which involve combining linguistic elements into more complex entities. Lexically based combinatorial processes typically combine morphemes into complex words through processes of derivation (*manage+ment=management*) or inflection (*jump+ed=jumped*), while syntactic combination involves combining words into syntactic phrases (e.g. noun phrases such as *the new book,* verb phrases such as *they walk carefully* or prepositional phrases such as *under the bricks*).

Patients with LH damage, especially involving LIFC, frequently have problems with syntax and inflectional morphology, both in production and in comprehension (Caplan & Futter 1986; Goodglass *et al*. 1993), even though the processing of simple, concrete words may remain relatively unimpaired. In a series of studies with such patients, we have shown that their comprehension of spoken inflected words (such as *blessed, jumped*) is significantly impaired (Marslen-Wilson & Tyler 1997, 1998; Tyler *et al*. 2002*a*,*b*; Longworth *et al*. 2005). These patients also typically have problems processing syntactic structure, although syntactic processing deficits are not confined to patients who only have damage to the LIFC. It is often difficult to attribute syntactic deficits solely to damage of the LIFC since damage to this region often also involves damage to proximate areas of the left superior temporal gyrus (STG). An important observation here is that patients who only have L posterior STG damage can also have problems with syntactic processing (Caplan & Hildebrant 1988; Caplan *et al*. 1996). We have observed the same kinds of behavioural deficits for syntactic processing in patients with intact LIFC but damage to L posterior STG/MTG, supporting the importance of both frontal and temporal cortex in spoken language function (see below and figures 11 and 12). Evidence from neuroimaging confirms the salience of LH contributions to combinatorial aspects of language function, primarily located in inferior frontal and temporal cortex, and in regions around inferior parietal cortex (Demonet *et al*. 1992; Zatorre *et al*. 1996; Binder *et al*. 2000; Friederici *et al*. 2003; Scott & Johnsrude 2003; Stamatakis *et al*. 2005; Tyler *et al*. 2005*c*), together with some involvement of the RH, albeit to a lesser extent.

But while there is broad agreement on these general aspects of spoken language processing, there is still considerable disagreement about the detailed properties of the neural language system and how different language processes are instantiated within it. Even very basic functions, such as the fundamental process of mapping speech sounds onto semantic representations, are not well understood, perhaps because few studies are underpinned by theoretical claims about the cognitive processes involved. Attempts to characterize the sound-meaning mapping range from the claim that it takes place within a hierarchically organized speech processing stream within the STG/STS, possibly bilaterally, with posterior regions engaged in the processing of form and anterior regions engaged in the processing of meaning (Scott *et al*. 2000; Scott & Johnsrude 2003), to the view that L posterior superior temporal regions play the crucial role, with the emphasis varying between Wernicke's area (BA 22; Hillis *et al*. 2001; Mesulam *et al*. 2003), the junction between L posterior temporal cortex and inferior parietal cortex (Mummery *et al*. 1999; Binder *et al*. 2000), and L posterior MTG (Dronkers *et al*. 2004; Indefrey & Cutler 2004). Still other researchers identify the L posterior middle and inferior temporal cortex as the critical site (Hickok & Poeppel 2004).

Similarly, the neural instantiation of other basic language processes, such as those underpinning syntactic and morphological analyses, remains unclear and controversial. Although there is convincing evidence that superior temporal cortex, possibly bilaterally, is engaged by processes involving morphological and syntactic combination (Friederici *et al*. 2003; Rodd *et al*. 2004; Tyler *et al*. 2005*b*), the role of other cortical regions in these processes is less certain. For example, while the LIFC, including Broca's area, is reliably activated when listeners are processing spoken language, its functions remain surprisingly contentious (see Kaan & Swaab (2002) for a review), despite its central role in most models of the neural language system since Paul Broca. On the one hand, sub-regions of LIFC are claimed to be functionally specialized for highly specific linguistic processes, such as syntactic parsing (Grodzinsky 2000; Friederici *et al*. 2003) or phonological analysis (Stromswold *et al*. 1996; Zatorre *et al*. 1996; Hagoort 2003), while on the other the LIFC is claimed to support general functions such as retrieval of linguistic information stored in posterior brain regions (e.g. Bokde *et al*. 2001; Gold & Buckner 2002), selection among competing alternatives (Thompson-Schill *et al*. 1997, 2005) or the maintenance of information in working memory (e.g. Gabrieli *et al*. 1998). Even the question of whether its role is specific to language is yet to be resolved, with several suggestions that LIFC supports processes shared by multiple cognitive domains (e.g. Miller 2000).

In summary, therefore, as this brief review indicates, there is still considerable uncertainty about the properties of the basic components of the neural language system, about the precise contribution of the regions themselves and how they operate together to support the dynamic processes of language comprehension and production. This state of affairs reflects, at least in part, the implicitly ‘phrenological’ assumptions underlying much current and historical research—namely a focus on delineating the functional specialization of specific brain areas, much as Broca and Wernicke originally attempted in their pioneering proposals nearly 150 years ago. To make progress, it is also necessary to focus on the nature of the functional relationships between the anatomically distinct regions which have been identified as constituting the neural language system, and how they are affected by different linguistic inputs. At the same time, however, it is necessary to do so in the context of a theory of the functional organization of the system as a cognitive process—in the current context, how speech inputs are mapped onto lexical representations and how these relate to processes of syntactic and semantic analysis. In the remainder of this paper, we outline recent research which attempts to address these issues.

In doing so, working with both intact and brain-damaged populations, we combine subtractive neuroimaging techniques with recent developments that provide analytic methods for examining the ways in which different regions within the neural language system interact with each other by analysing functional connectivity between cortical regions. This enables us to go beyond a description of the neural language system in terms of levels of activity in isolated regions, by determining the ways in which activity in one region modulates activity within another. These analyses capture time-dependent changes in the coupling or decoupling of anatomically remote brain areas, allowing us to study integration in the brain in the context of changing task conditions in a dynamic manner (Fletcher *et al*. 1999). We have exploited this type of approach, in combination with more conventional subtractive analyses of neuroimaging data, in order to determine the nature of the interactions between different cortical regions with respect to two core sets of linguistic functions, covering combinatorial operations in the lexical and in the grammatical domains.

## 2. Functional organization of the fronto-temporal language system: words and morphemes

We focus first on the lexical domain, examining the representational and processing consequences of inflectionally complex words such as *jumped* or *smiles*. These are forms, very common in English, made up of a noun or a verb stem and an inflectional morpheme (‘jump’+‘-ed’) or (‘smile’+‘-s’). Inflectional (or grammatical) morphemes are particularly revealing because they link across the two fundamental combinatorial domains of lexical and syntactic combination. Forms like *jumped* or *smiles*, reflecting the operations of regular inflectional morphology in English, on the one hand engage and challenge the basic systems of lexical access, whereby phonological forms are mapped onto internal representations of lexical form and meaning. On the other hand, the information carried by these forms—especially the inflectional morphemes themselves—has a critical role to play in combining incoming words and morphemes into higher order structures. We therefore begin this analysis of functional connectivity by examining the processing of regularly inflected verbs and nouns. These enable us to probe basic processes of lexical access, the morpho-phonological parsing operations required by inflected forms and the structural processes that depend on the information carried by grammatical morphemes. This set of processes implicates key temporal and frontal lobe structures in these basic linguistic operations.

In considering these processes, we need to take the *morpheme*—the minimal meaning bearing element in human language—as a basic building block. Broadly speaking, morphemes can either carry semantic content or can function to communicate grammatical information of various types. In a language like English, semantic morphemes can almost always occur as monomorphemic ‘free stems’—as in words such as *dog*, *smile*, *tidy*, etc.—whereas grammatical morphemes are often ‘bound’ and only occur in conjunction with semantic morphemes. The plural morpheme {-s}, for example, can only appear in combination with a free stem such as {dog}, creating the form *dogs*. Similar constraints hold for the past tense morpheme {-d}, in forms like *smiled* or *jumped*, as well as for a large range of derivational morphemes (e.g. {-ness} combining with {tidy} to create the form *tidiness*).

Consistent with extensive neuropsychological evidence and at least some neuroimaging evidence, we assume that lexical access processes involving monomorphemic content words—the initial mapping of acoustic–phonetic information in the speech signal onto stored lexical representations of form and meaning—are mediated by brain regions in the superior and middle temporal lobes. As noted earlier, these lexical access processes seem to be supported bilaterally, although there is undoubtedly some degree of LH dominance. An increasing body of data, from both neuropsychological and neuroimaging sources, indicates that morphologically complex words involving regular inflectional morphology require these temporal lobe access processes to interact with inferior frontal areas, primarily via a so-called ‘dorsal’ route involving the arcuate fasciculus, likely to be critical for morpho-phonological parsing.

Perhaps the most direct evidence for the involvement of this dorsal route—as opposed to the ventral route likely to be active in more semantic aspects of language comprehension (as discussed later)—comes from a recent study using a lesion–behaviour correlational technique (Tyler *et al*. 2005*a*). This is a new methodology which correlates scores on two continuous variables—signal intensity of each voxel across the entire brain of brain-damaged patients and their continuous behavioural scores, in this case from a priming study. We used this method to determine whether disruption to the processing of regularly or irregularly inflected past tense forms is associated with damage to different brain regions. We correlated signal intensity across the brains of 22 right-handed brain-damaged patients, with the patients' behavioural scores on a priming study which tested their ability to process the phonological form, meaning and morphological structure of spoken words.

In the priming study, patients heard prime–target stimulus pairs and made a lexical decision to the second (target) stimulus in each pair. We compared word-pairs which were either regularly inflected past tense forms (e.g. *jumped-jump*) or irregularly inflected past tense forms (*slept-sleep)*, or related only in phonological form (e.g. *pillow-pill*) or only in meaning (e.g. *card-paper*). Different neural regions correlated with behavioural priming scores in the four conditions. Priming for regularly inflected past tense words correlated most strongly with variations in signal intensity in the LIFG (BA 47, 45), as shown in figure 2*a*. At a slightly lower threshold (figure 2*b*), this cluster included a large region of left superior temporal gyrus (LSTG) extending from the anterior extent of Wernicke's area (BA 41, 42) to the anterior LSTG. When the threshold was lowered still further (figure 2*c*), all of Wernicke's area was included, looping around to include the arcuate fasciculus and Brodmann areas 47, 44 and 45 (Broca's area). This essentially replicates the classical Broca–Wernicke–Lichtheim model of language function, where the arcuate fasciculus connects superior temporal and inferior frontal regions in a neural language system (figure 1), and is similar to the dorsal route identified in more recent neural accounts of the language system (e.g. Hickok & Poeppel 2000).

Priming for the irregularly inflected past tense forms, in contrast, correlated with signal intensity in completely different neural regions—the left superior parietal lobule, inferior parietal lobule and angular gyrus. These regions have been associated with irregular past tense processing in previous neuroimaging studies (Jaeger *et al*. 1996; Beretta *et al*. 2003) and are often reported as being activated in lexical processing tasks (Demonet *et al*. 1992; Celsis *et al*. 1999). The role of these regions in lexical processing is confirmed by the finding that when they are damaged, which typically occurs following lesions in Wernicke's area, the patient exhibits the speech comprehension deficits typical of Wernicke's aphasia (Selnes *et al*. 1985; Kertesz *et al*. 1993). Wernicke's area and the surrounding parietal regions are thought to be involved in the mapping between spoken forms and their meanings.

In a further series of behavioural and neuroimaging experiments, we used the contrast between words with regular and irregular past tense morphology to build up a more dynamic picture of the fronto-temporal systems underlying combinatorial morphological processes involving inflectional morphemes. The value of this contrast is that it provides sets of words that are matched for lexical and grammatical properties, but which differ in whether or not they are decomposable, via morpho-phonological parsing processes, into a stem morpheme plus an inflectional affix. This always holds for regularly inflected forms, like *smiled* or *jumped*, but not for irregular past tense forms, such as *bought* or *gave*, which cannot be decomposed into a stem plus an affix, and where the idiosyncratic and unpredictable nature of each form means that they have to be learnt and stored as unanalysable whole forms.

The importance of morpho-phonological parsing processes in the perceptual analysis of regular inflectional morphology was highlighted in a previous study with brain-damaged patients, where we found that patients with left perisylvian lesions had difficulties in processing stimuli (whether real words or non-words) that ended in either real or potential inflectional morphemes (Tyler *et al*. 2002*a*,*b*). These patients were impaired not only on regularly inflected past tense forms such as *played*, but also on real words like *trade* and non-words like *snade* which shared the specific phonological features that are diagnostic of the presence of a potential inflectional suffix. These features—a word-final coronal consonant (typically /d,t,s,z/) that agrees in voice with the preceding segment—we refer to as the *inflectional rhyme pattern*. The fact that the patients were impaired on all three types of words, but had no difficulty in processing words which did not contain these phonological properties, suggested that the processes of morpho-phonological parsing were disrupted in these patients because of damage to their L perisylvian language areas. These were patients where processing of monosyllabic content words was generally spared, based on relatively intact temporal lobe systems (LH and/or RH), but where the fronto-temporal system linking basic lexical access with combinatorial morpho-syntactic processing was in some way disrupted.

These hypotheses about patient performance made clear predictions about how the presence or absence of morpho-phonological complexity should affect the distribution of neural activity in the intact brain. We pursued these predictions in an fMRI study with healthy subjects (Tyler *et al*. 2005*c*), following this up with a re-analysis of the same data in a functional connectivity framework (Stamatakis *et al*. 2005). Listeners heard spoken word-pairs, such as *played-play* or *played-played*, and indicated, by means of a button-press, whether the pairs were the same or different. The same three types of real and pseudo-inflected forms were used (*played*, *trade* and *snade*), all sharing the inflectional rhyme pattern, as well as a matched set of real and non-word pseudo-irregulars (*thought*, *port* and *hort*), which do not end in potential suffixes. These were embedded in same–different pairs such as *thought-think*, *thought-thought*, etc. A third set consisted of simple words which have no morphological structure (e.g. *shelf-shell*) and which also did not end in potential suffixes. However, these pairs were similar to the regulars in sharing the same minimal (one phoneme) difference between word-pairs, controlling for the possibility that differential effects for the regulars might simply reflect the difficulty of making the same–different decision between stimuli which are perceptually very similar. If the neural language system is differentially sensitive to phonological cues which signal morphological decomposition, then we would expect a different pattern of activation for the regularly inflected sets compared with either the irregularly inflected or the simple sets, neither of which can be decomposed and must be accessed as full forms.

The fMRI analyses (Tyler *et al*. 2005*c*) showed that stimuli containing phonological cues to the presence of a potential suffix preferentially activated a fronto-temporal network, including anterior cingulate cortex (ACC), LIFG, bilateral STG, L inferior parietal lobule (LIPL) and bilateral MTG, over and above activation for the irregular sets (figures 3*a*,*b*) or the simple words (figure 3*c*). There were no regions that were significantly more activated for the irregulars when compared with the regulars, or for the simple words when compared with the regulars. This increased LIFG activation arguably reflects additional processes of morpho-syntactic analysis which are required for parsing regularly inflected forms into their stems and morphological affix. The finding that LIFG activation was obtained for inflected non-words as well as for real words, suggests that it is the morpho-phonological structure (real or potential) of stimuli containing the inflectional rhyme pattern that produces the additional activation. Further evidence for this comes from a comparison between two sets of non-words—regular non-words (e.g. *crade-cray*) and simple non-words (e.g. *blane-blay*). This contrast showed an increased activation for the regular non-words when compared with the simple non-words but only in the LIFG and not in the LMTG or STG. When neither a stem nor a whole word is accessed (as is the case for non-words), then there is no differential STG/MTG activity, but the inflectional rhyme pattern present in the regular non-words still triggers the LIFG.

We attribute the increased LMTG and STG activation (figure 3) for real regular and pseudo-inflected forms to the special processing demands made by such forms. Although *jump*, or any other uninflected stem, can map straightforwardly onto temporal lobe lexical representations, the presence of a past tense affix, as in the form *jumped*, seems to place additional demands on this access process. To interpret *jumped* correctly, and to allow the process of lexical access to proceed normally, the recognition system requires the simultaneous access of the lexical content associated with the stem *play* and of the grammatical implications of the {-d} morpheme. This seems to require both an intact LIFC and intact links to left superior temporal cortex. Note that irregular past tense forms, which are never realized as an unchanged stem plus an affix, are not subject to the same additional processing requirement. They are assumed to be accessed as whole forms, exploiting the same temporal lobe systems as uninflected stems. Although irregular past tense forms will activate LIFC to some extent, owing to their morpho-syntactic implications, immediate access to their lexical meaning does not obligatorily require LIFC phonological parsing functions in the same way as regular past tense forms.

Independent evidence for this functional analysis comes from recent priming results (Longworth *et al*. 2005) demonstrating that patients with LIFC damage and difficulties with regular inflectional morphology also show deficits in semantic priming when the primes are regularly inflected forms, as in pairs like *jumped*/*leap*. At the same time, critically, they show normal performance both for pairs with stems as primes, as in *jump*/*leap*, and for pairs where the prime is an irregular past tense form, as in *shook*/*tremble*. Normal semantic priming performance in these auditory–auditory paired priming tasks requires rapid access to lexical semantic representations in the processing of both prime and target. The patients' preserved performance for stem and irregular spoken primes shows that the systems supporting fast access of meaning from speech are still intact for these types of input. The decrement in performance on the regular inflected forms means that these inflected forms make special processing demands and that an intact LIFC (and intact dorsal fronto-temporal links) are necessary to meet these demands.

A key component of this account is the claim that these special processing demands are automatically elicited by any input that shares the diagnostic properties of an inflectional affix, whether or not these forms correspond to existing lexical representations. Unless the system attempts the morpho-phonological segmentation of forms like *trade* or *snade*, it cannot rule out the possibility either that the pseudo-regular *trade* is actually the real regular *tray* in the past tense or that *snade* is the past tense of the potentially real stem *snay*. This, we argue, requires obligatory access to left inferior frontal regions. Additional evidence to support the across-the-board impact of the inflectional rhyme pattern comes from a recent behavioural study with intact young adults (Post *et al*. 2004), which not only replicates the finding that real, pseudo and non-word regulars group together against a range of control conditions, but also suggests that similar contrasts apply to English {-s} inflections, as in *jumps* or *yards*, which obey the same constraints of coronality and agreement in voice.

In summary, therefore, the increased activation for regulars (and pseudo-regulars) in temporal and inferior frontal areas reflects, on the one hand the specialized role of LIFC processes involved in analysing grammatical morphemes, and on the other the continuing STG/MTG activity involved in accessing lexical representations from the stems of regular and pseudo-regular inflected forms. The LIFC functions invoked here are likely to include support both for morpho-phonological parsing, segmenting complex forms into stems and affixes, and for syntactic processes triggered by the presence of grammatical morphemes such as the past tense marker.

A final consideration here is the potential control processes which regulate the proposed processing relationship between L frontal and temporal regions. Several lines of evidence suggest that the integration of information between superior temporal and L frontal areas may be modulated by anterior midline structures including the anterior cingulate, which both neuroanatomical and functional neuroimaging evidence suggest is well suited for this role. Work with non-human primates shows that the ACC projects to or receives connections from most regions of frontal cortex (Barbas 1995) and from superior temporal cortex (Pandya *et al*. 1981). Recent neuroimaging data not only implicate the ACC in the modulation of fronto-temporal integration (Fletcher *et al*. 1999), but also show it to be active in situations requiring the monitoring of interactions between different information processing pathways (Braver *et al*. 2001).

In this view, the increased activation of the ACC by real regular inflected forms (figure 3*b*) may reflect the greater demands made on this monitoring function when complex forms such as *jumped* need to be parsed into a stem plus affix, with the bare stem then being able to act as a well-formed input to STG lexical access processes. The nature of this potential ACC contribution is examined in more detail in the connectivity analyses described below.

## 3. Functional connectivity in the intact and damaged brain: words and morphemes

The research described so far provides evidence for the activity of both frontal and temporal structures in the processing of morpho-phonologically complex words, combining evidence from behavioural studies with intact and brain-damaged populations with neuroimaging studies of the intact adult brain. We followed up the subtractive analyses reported above (figure 3) with a series of connectivity analyses on the same data, in order to address more directly the functional relationship between the regions within the fronto-temporal language system. To do so, we used an approach which extended earlier proposals of Friston *et al*. (1997), aimed at identifying how the covariance between two regions could be modulated by a psychological variable or alternatively by the level of activity in a third region. The former was referred to as a psycho–physiological interaction and the latter as a physio–physiological interaction. In our study, we extended this approach to include both the psychological variable (morphological complexity) and the activity in a third region. This is therefore referred to as a psycho–physio–physiological interaction (Stamatakis *et al*. 2005).

The resulting connectivity analysis (figure 4) shows that the LH regions identified in the subtractive analyses (figures 3*a*,*b*), in LIFC and ACC, predict activity in L posterior middle temporal gyrus (MTG) for regularly inflected forms when compared with irregularly inflected forms (figure 4*a*)—for example, *played* versus *taught*. A comparable analysis carried out on RH activations showed that the RACC and RIFC strongly predicted activity in LMTG (figure 4*c*) and, to a lesser extent, in RMTG (figure 4*b*).

This fronto-temporal interaction was reduced when the words were phonologically similar to the regular and irregular past tense but not themselves morphologically complex (e.g. for contrasts like *trade* versus *port*), suggesting that the modulatory effects we found for the regulars reflect the greater integration of the fronto-temporal language system required for processes of morpho-phonological decomposition and analysis rather than phonological differences between the regulars and the irregulars. The greater activation for real as opposed to pseudo-regulars reflects the likelihood that a form like *played* will trigger more activity than *trade,* both in terms of its consequences for the lexical access process and in terms of morpho-syntactic analysis processes. These latter processes will presumably be engaged more strongly when the evidence suggests that a grammatical morpheme is indeed present.

These results, showing connectivity between inferior frontal and middle temporal regions, are consistent with anatomical connectivity via the arcuate fasciculus between frontal and temporal regions, and between orbito-frontal and anterior temporal regions via ventral connections (Petrides & Pandya 1988; Morris *et al*. 1999). They are also consistent with recent analyses of the anatomical connections in the human brain, using DTI (Catani *et al*. 2005; Parker *et al*. 2005). As noted earlier, this work suggests that there may be important asymmetries in the anatomical connectivity between the cortical regions implicated in language function in the R and LH. In the DTI analyses of Parker *et al*. (2005), it is only in the LH that there is clear evidence for both a dorsal route, connecting Wernicke's and Broca's areas via the arcuate fasciculus, and a ventral route, connecting the LMTG to the LIFG via the uncinate fasciculus.

Connectivity studies with normal populations, as described above, form a valuable basis for investigating and interpreting the consequences of damage to the brain systems in question. Neuroimaging studies of patients with damage to the LH fronto-temporal system but preserved RH fronto-temporal cortex provide important additional information about the regions which are necessary for processing different types of linguistic inputs. Since, on the basis of the data described above, we claim that co-activation and modulation of LH fronto-temporal systems is integral to the processing of regularly inflected words, damage to this system should lead to greater difficulty in processing regularly when compared with irregularly inflected past tense forms. This should be revealed in abnormal patterns of L fronto-temporal connectivity.

To evaluate this hypothesis, we recently ran a chronic aphasic patient in the fMRI study described above (Tyler *et al*. 2005*c*). This patient, labelled here as patient P1, had extensive L perisylvian damage and showed persistent difficulties with the regular past tense which generalized to any speech token containing the diagnostic features of the inflectional rhyme pattern (Tyler *et al*. 2002*a*,*b*). In contrast to healthy age-matched controls, who showed increased activation for regular compared with irregular inflected forms in LIFG and bilateral STG/MTG (similar to the young normal patterns in figure 3), P1 showed greater activity for the regulars in the RIFG and in the R insula. Note that the LMTG activations from the connectivity analysis carried out on unimpaired subjects (figure 4*a*), which were predicted by the combined effects of the LIFG and the LACC, fell into damaged regions in the patient (figure 5*c*). Owing to his extensive LH damage, connectivity analysis could only be carried out on the RH for this patient.

In order to compare P1 with the appropriate control group, we first carried out a connectivity analysis on a small group of healthy subjects age-matched to the patient. Figure 5 shows the pattern of connectivity for this group, which generated essentially the same pattern of connectivity as the young with one exception; the LIFG and LACC predicted activity in RMTG as well as LMTG (figures 5*a*,*b*), perhaps indicating a degree of hemispheric reorganization with increasing age. The patient's connectivity analysis showed a stronger RH pattern of connectivity when compared with controls (figure 5*c*) with increased activity in R inferior and RMTG, as well as the anterior temporal lobes in both the hemispheres (figure 5*c*).

These anterior temporal areas are typically associated with semantic processing, consistent with such patients' greater reliance on semantic and pragmatic factors in language processing. P1's behavioural deficit in processing regularly inflected words (Tyler *et al*. 2002*a*,*b*; Longworth *et al*. 2005), and with syntax more generally (Tyler 1992), coupled with his extensive L perisylvian lesion and abnormal connectivity analyses, underlines both the importance of the LIFC and the apparent inability of the RIFC to take over the functions of the LIFC in these core domains of normal language processing. The RIFC activation that we observe for the patient, and its associated connectivity, may reflect functional reorganization where the patient evolves alternative strategies to meet the demands of language comprehension, building on residual functions of the normal network. This reorganization, nonetheless, notably fails to support the on-line processes of morpho-phonological segmentation and morpho-syntactic analysis required for successful processing of inflectionally complex words.

## 4. Functional organization in the intact and damaged brain: syntax and semantics

So far, our focus has been on how activity within the fronto-temporal language system is modulated as a function of the linguistic properties of individual words. The essence of language comprehension, however, involves combining words into structured sequences through processes of syntactic combination. Although the LIFG has long been considered to play a prominent role in these processes, there is a continuing disagreement about the nature of its contribution. At one extreme is the view that the LIFG supports general cognitive functions such as working memory and selection and is not specialized at all for syntactic processing (Thompson-Schill *et al*. 1997; Gabrieli *et al*. 1998; Miller 2000; Kaan & Swaab 2002). On the other hand, the LIFG is claimed to have a key role in syntactic processing, with Friederici (2004), for example, claiming that BA 44/45 in LIFG is involved in hierarchical structure-building, needed to capture long-distance dependencies between words and phrases, while phrasal level syntactic analyses—such as combining words into noun (e.g. *the dog*) and verb phrases (e.g. *he runs*)—involve the L frontal operculum (medial to BA 44). In contrast, Hagoort (2003) argues that the L posterior temporal cortex is important for the retrieval of syntactic frames stored in the lexicon whereas the LIFG binds this and other types of lexical information (phonology and semantics) together.

Accompanying these uncertainties about the nature of LIFG contributions is an equal degree of uncertainty about its relationship to other brain regions supporting language function, especially in the temporal lobes, as well as about the precise contribution that these regions themselves make to processes of language comprehension. While it is plausible that major dorsal and ventral processing streams, linking auditory processing areas in STG/STS to temporal, parietal and frontal regions, are involved in syntactic and sentential analyses (Hickok & Poeppel 2004), there are basic disagreements about the functional characterization of these pathways. In most accounts, the functional relationship between frontal and temporal areas is unspecified, and little attention is paid to the properties of parallel regions in the RH.

Our approach to these issues has been to explore, through connectivity analyses on fMRI data from both unimpaired and brain-damaged patients, the processing dependencies between frontal and temporal regions during the processing of spoken sentences. To understand LIFG function in the context of language processing requires an understanding of the functions it performs relative to the processing functions of other components of the neural language system.

To investigate the relationship between fronto-temporal systems in processing syntactic structure, we have carried out fMRI studies which differentiate semantic and syntactic sentential processing. In one recent study, we did this by presenting listeners with spoken sentences containing either semantic or syntactic ambiguities (Rodd *et al*. 2004). Ambiguity is a natural aspect of language; it occurs frequently and is rarely noticed by listeners because it is typically resolved almost immediately by the presence of a disambiguating context. For example, in ‘She quickly learnt that injured calves…’, the word *calves* has more than one meaning and is therefore momentarily ambiguous. However, this ambiguity is disambiguated by the following words ‘…moo loudly’. Sentences can also contain phrases which are syntactically ambiguous. For example, in the sentence ‘Out in the open, flying kites…’, ‘flying kites’ is syntactically ambiguous in that either *flying kites* can be a noun phrase in which *flying* modifies the noun *kite* or a verb phrase where flying is a progressive participle (as in ‘I was flying kites’). This ambiguity can be immediately resolved by the inflection on the subsequent verb (e.g. ‘… *are*’/‘ …*is*’; Tyler & Marslen-Wilson 1977). Moreover, ambiguity is not a binary variable; words and sentences can vary in the degree to which they are ambiguous. We factored this into our study by obtaining ‘dominance’ ratings for each ambiguity. These provided an estimate of the extent to which one reading of a semantically ambiguous word or syntactically ambiguous phrase was preferred by listeners and were entered into the imaging analysis.

Using ambiguity as a way of manipulating syntactic and semantic structures avoids the criticisms that have been levied against previous studies, by minimizing overt working memory demands (Kaan & Swaab 2002). To reduce task requirements still further, we used a task which had been shown previously to produce patterns of activation which are indistinguishable from passive listening (Rodd *et al*. 2005). Listeners heard spoken sentences, and at the end of the sentence saw a visually presented probe word and made a judgement, indicated by a button-press, as to whether the word was related to the meaning of the sentence.

Syntactic ambiguity produced increased activation in LIFG (BA 44, 45, 47) and in a large swathe of LMTG, extending anteriorly into the anterior STG and posteriorly to the inferior parietal lobule (figure 6). There was also a smaller cluster of activation which included the RSTG (Rodd *et al*. 2004). Activation in these regions increased as a function of increasing dominance, such that they were more strongly activated when the ambiguous phrase was followed by a continuation which was inconsistent with the strongly preferred syntactic interpretation. These regions are increasingly involved when listeners develop strong preferences for one particular syntactic reading, which is then overturned by the subsequent input, forcing a reinterpretation of the syntactic structure. Semantic ambiguity activated a subset of the same fronto-temporal regions as syntactic ambiguity. Although the LIFG activity overlapped considerably for both types of ambiguity, the LMTG activation for semantic ambiguity was confined to the mid portion of the MTG and did not extend posteriorly. Moreover, activity in the LMTG was substantially less than for syntactic processing and was only significant at a slightly lower threshold (figure 6). In addition, the effect of semantic ambiguity was unaffected by the extent to which one meaning of a word was more strongly preferred over another, suggesting that both meanings are activated and listeners wait to make their choice until they hear the disambiguating information.

These results suggest that different cognitive strategies, seemingly rooted in separable underlying processing systems, govern the processing of the syntactic and semantic aspects of sentences. Younger listeners appear to handle syntactic ambiguity by choosing the most frequent reading and revising this interpretation when it fails to match the subsequent input. In contrast, they appear to delay their commitment to either reading of a semantic ambiguity until they have confirmatory information. These different sets of analysis processes affect the neural language system differentially, with only syntactic analysis engaging posterior temporal/parietal regions in the LH, perhaps indicating its particular involvement in combinatorial processing when working in concert with the LIFG.

## 5. Functional connectivity in the intact and damaged brain: syntax and semantics

Functional connectivity analyses can further sharpen these potential contrasts in the processing relationship between frontal and temporal cortices for syntactic and semantic aspects of sentential analysis. To explore this, we compared the activation patterns for sentences containing syntactic and semantic ambiguities with matched unambiguous sentences, using the peak frontal activations from the relevant subtractive analyses to predict activity elsewhere in the brain. The resulting functional connectivity analyses reveal distinct patterns of fronto-temporal connectivity for the two types of linguistic computation. For semantic processing (as shown in figure 7*a*), activity in the LIFG positively predicts activation in the L temporal pole (BA 38), suggesting that this region and the LIFG co-modulate each other's activity during semantic processing.

The syntactic functional connectivity analysis (figure 7*b*) showed the same co-modulation between LIFG and L temporal pole as for semantic processing, which is not surprising given that all sentences involved semantic analysis. However, in the syntactic analysis, this anterior STG activity was bilateral. Moreover, for the syntactic analysis only, the LIFG also predicts activity in LH posterior regions which included the L posterior MTG, L inferior parietal, angular gyrus and supramarginal gyrus (figure 7*b*). These results suggest that syntactic combinatorial processes, revealed most strongly when the process is disrupted, involve the co-modulation of LIFG, bilateral anterior STG and left posterior temporal–parietal sites.

The left temporal areas that are active in these analyses of syntactic activity turn out to be adjacent to, but not overlapping with, the L posterior MTG region that showed a greater connectivity with the LIFG for regular when compared with irregular inflected words (figure 8). The fact that activity in the LIFG during semantic processing is not correlated with activity in these more posterior temporal regions, whereas syntactic and morpho-phonological processing does seem to be, invites the inference that these adjacent regions of left posterior temporal cortex play related but different roles in mediating combinatorial linguistic processes.

Overall, these functional connectivity results suggest that successful syntactic processing requires the joint activity of an intact network of LH regions including the LIFC and regions of posterior temporal and parietal cortex. In contrast, semantic processing, while also involving the LIFC, engages a more anterior region of the LMTG/STG. Given these results, lesions which include LIFG and/or posterior temporal–parietal regions would be expected to impair syntactic processing. In a preliminary test of this hypothesis, using both subtractive neuroimaging methods and functional connectivity analyses, we studied two illustrative brain-damaged patients. One of these (patient P1) had extensive LIFC damage as well as damage which extended into temporal perisylvian language regions (figure 5*c*), whereas the other (patient P2) had an intact LIFC but a lesion in L posterior temporal cortex, mostly involving the MTG (figure 9*a*). Both had well-documented difficulties with syntactic processing in a variety of different tasks, while semantic processing was unimpaired (Tyler 2002*a*).

Patient P1 showed an abnormal pattern of activity for syntactic ambiguity, consistent with his behavioural deficit (figure 9*a*). Syntactic ambiguities, when compared with unambiguous sentences, produced substantial perilesional activity in the L middle frontal gyrus and pre- and post-central gyrus, and in the right inferior parietal lobule, a region slightly more posterior than the comparable activations in the LH in healthy listeners. Connectivity analyses using the peak voxels in the LH from the subtractive analysis predicted activity in R posterior regions, including the R angular gyrus, supramarginal gyrus and inferior parietal lobule (figure 9*b*). This anomalous network must reflect some degree of functional reorganization, given the destruction in this patient of so much of the left perisylvian network that supports syntactic function in the unimpaired brain. However, although this substitute sub-system seems capable of supporting some aspects of syntactic analysis—otherwise effects of syntactic ambiguity would not have been elicited—it is clearly unable to restore the key combinatorial functions underpinning normal performance.

For the same patient, semantic ambiguity extensively activated right frontal and bilateral parietal regions (figure 10*a*), with the largest cluster in the RIFG. The exceptional extent of these activations may itself reflect another form of functional adaptation in this patient. Because normal syntactic constraints are not available, the processes of speech comprehension in such patients are heavily dependent on the semantic and pragmatic properties of the input. This means that processing is particularly strongly disrupted when these semantic expectations are violated, as we saw in earlier behavioural experiments (Tyler 1992) when this patient encountered semantic violations, as in ‘John drank the guitar’. The functional connectivity analysis (figure 10*b*) showed that activity in the RIFG predicted activation in the L posterior MTG and also in R anterior STG (figure 10*b*), in regions similar to those activated in healthy subjects (figure 7*a*), although here the LH anterior temporal activation is not seen. Given this relatively normal pattern and that this patient does not have a semantic deficit, it is clear that language-related semantic processing can be achieved by means of a more distributed, more bilateral fronto-temporal system than is the case for syntax and does not seem to be dependent on the input from intact left perisylvian language areas to the same extent as syntactic processing.

Turning to patient P2, with damage restricted to L posterior temporal areas (figure 11*a*), and with no LIFG involvement, here syntactically ambiguous sentences produced greater activation in the RIFC rather than the LIFC, even though the LIFC was not damaged. The fact that activation in the RIFC was nonetheless accompanied by a syntactic deficit is consistent with the view that the RIFC cannot play the same functional role as the LIFC in syntactic processing. In contrast, semantically ambiguous sentences produced a pattern in this patient similar to that of healthy subjects, with peak activation in the LIFC. We then carried out functional connectivity analyses on these data, using the peak activations from the subtractive analysis. Note that in the absence of significant LIFG activation in the syntactic conditions, these syntactic connectivity analyses are driven by seeds in the RIFG (figure 11*c*).

In the semantic condition (figure 11*b*), activity in the LIFG predicted activity in anterior LSTG/MTG (BA 21), a region close to that activated for healthy subjects (figure 7*a*), as well as in the RSTG. In the syntactic analysis (figure 11*c*), this same L anterior STG/LMTG region was modulated by activity in the RIFG. The RIFG also positively predicted activity in bilateral posterior STG/MTG and IPL. The posterior LH activity was just perilesional to the patient's damage. These results suggest a degree of reorganization of function. Unlike in healthy subjects, semantic processing, which appears to be unimpaired, involves the co-activation of the LIFC and bilateral temporal cortex. Syntactic processing also involves a more bilateral system of connectivity than healthy subjects, with posterior temporal-parietal activity in the RH as well as in the LH, although with no LIFG activity detected. In spite of this additional RH involvement, syntactic processing is impaired, again consistent with the observation that this region cannot fully compensate for damage to critical LH regions and their connectivity (see below).

The patient's connectivity analysis reveals an abnormal pattern of connectivity for syntactic processing, which is associated with an abnormal behavioural profile. In contrast, semantic processing is normal, in terms of both functional connectivity and behaviour. We can unpack these contrasts still further, using recent developments in neuroimaging techniques, to ask whether the patient's syntactic deficit was due solely to grey matter damage in left posterior temporal cortex or whether white matter tracts connecting this region to other regions within the neural language system were also compromised. This is an important issue because patients with damage to posterior temporal cortex differ in the nature of their language deficits, with some showing evidence of a syntactic deficit and others not (Zurif *et al*. 1993; Wilson & Saygin 2004). One possible explanation for variation in the effect of left posterior temporal lesions may lie in the extent to which damage compromises the white matter connections between the lesion site and other anatomically distributed regions of the language system. Given that syntactic processing involves both posterior temporal and inferior frontal regions, syntactic deficits may be restricted to those patients whose damage includes the white matter tracts connecting these regions.

To determine whether there were any abnormalities in the patient's white matter tracts, we obtained DTI data and calculated fractional anisotrophy (FA), which provides a measure of the integrity of white matter tracts *in vivo,* by measuring directionality of water diffusion in each voxel (Basser & Pierpaoli 1996). In this analysis, we were primarily interested in the major white matter tracts which are thought to be of special importance in language function—the dorsal running arcuate fasciculus and the ventral running inferior longitudinal fasciculus—and therefore confined our analyses to these regions.

Figure 12 shows FA maps for patient P2 and, for comparison purposes, a healthy subject of a similar age. As the figure shows, the integrity of the patient's white matter tracts differs markedly across the hemispheres, with greater integrity in the RH than in the LH. Comparing the FA values in the arcuate and inferior longitudinal fasciculi in the two hemispheres confirmed this pattern for the patient. The mean FA for the patient in the LH was 0.235, whereas it averaged 0.349 in the RH. In contrast, for the age-matched healthy control, there was no difference across the hemispheres, with FA averaging 0.377 in the LH and 0.367 in the RH. Moreover, when compared with the control subject, the patient showed a greater reduction in FA in the arcuate fasciculus than in the inferior longitudinal fasciculus. In fact, as figure 12 shows (indicated by the white arrow), there is an apparent discontinuity in the left arcuate fasciculus which is neither present in the patient's RH nor in the healthy control.

This is an important observation for several reasons. First, it invites the inference that the disruption of this route between frontal and posterior temporal regions is a critical factor in the syntactic deficit shown by this patient. Second, it reinforces the significant theoretical and clinical point that the functional deficits associated with damage in particular locations needs to take into account white matter as well as grey matter damage. Finally, it underscores the critical role of connectivity between brain regions in characterizing the neural substrate for core linguistic functions.

## 6. Overview and conclusions

The research described in the preceding sections combines psycholinguistically well-motivated questions about different aspects of human language comprehension with behavioural and neuroimaging studies of normal performance, incorporating both subtractive analysis techniques and functional connectivity methods, and applying these same tasks and techniques to the analysis of the functional and neural properties of brain-damaged patients with selective linguistic deficits in the relevant domains.

The results of these investigations point to a set of partially dissociable sub-systems supporting three major aspects of spoken language comprehension, involving regular inflectional morphology, sentence-level syntactic analysis and sentence-level semantic interpretation. Differential patterns of fronto-temporal connectivity for these three domains confirm that the core aspects of language processing are carried out in a fronto-temporo-parietal language system which is modulated in different ways as a function of different linguistic processing requirements. No one region or sub-region holds the key to a specific language function; rather each requires the co-activation of activity within a number of different regions.

The use of functional connectivity analyses, in both intact and impaired systems, is critical to the ability to tease apart the wealth of overlapping activity associated with each function. While standard subtractive analyses delineate a range of regions potentially involved, functional connectivity analysis plays the critical role of indicating which regions directly participate in a given sub-process, by virtue of their joint time-dependent activity. By revealing these codependencies, connectivity analysis sharpens the pattern of structure–function relations underlying specific aspects of language performance.

Within the three aspects of language function addressed here, two of these, involving inflectional morphological and syntactic processes, clearly group together in distinction from the third, semantic function. Where the latter is concerned, the most salient outcome is the robustness of the ability to construct a semantic interpretation from linguistic inputs, even in the face of massive disruption to core LH language areas. A patient like P1 is able to use lexically derived semantic and pragmatic cues to meaning to drive an effective on-line interpretation process, with normal performance in semantic priming tasks (as long as inflectional morphology is not involved), and with normal sensitivity to semantic and pragmatic constraints in the speech input (e.g. Tyler 1992; Longworth *et al*. 2005). Functional connectivity analyses for P1 show considerable reorganization of functional networks, with additional recruitment of anterior temporal areas related to semantic function in the processing of isolated words (figure 5*c*), and with greatly increased RH involvement in sentence-related semantic processing (figure 10). Patient P2, with disruption of LH syntactic function, nonetheless shows normal performance on semantic tasks, in the context of stronger bilateral involvement (figure 11*b*). Damage in other brain areas may well produce permanent impairment in semantic function, but for patients with L perisylvian damage it is clearly possible to retain, and perhaps to rebuild, the ability to semantically interpret spoken utterances, on the basis of functional reorganization of the neural substrates involved.

Both morphological and syntactic processes, in contrast, require an intact left-perisylvian language system—perhaps because they share a core language-specific combinatorial element (however this might be realized neuro-computationally). If the key LH regions (or the connections between them) are damaged, then the system seems to be unable to reorganize to restore effective morphological or syntactic function. Both P1 and P2 provide evidence for some degree of stable reorganization, with novel combinations of regions co-active in response to syntactic processing demands, but this had little impact on their continuing syntactic deficits.

Despite these core similarities, however, there are also substantial differences in the neural sub-systems linked together to support inflectional morphological processes on the one hand, and clausal and sentence-level syntactic interpretation on the other. The key network identified for regular inflectional morphology is relatively compact, and links LIFG, ACC and an area in L MTG (figure 4*a*). This LMTG activation, likely to be implicated in basic lexical access processes (Dronkers *et al*. 2004), is adjacent to, but distinct from the more posterior temporo-parietal regions activated in the syntactic functional connectivity analyses (figures 7*b* and 8), which extend into the L supramarginal gyrus, the angular gyrus and the IPL. The network implicated in syntactic processing also links to substantial areas of activation in bilateral anterior MTG/STG, showing some overlap with areas implicated in semantic processing (figure 7), and presumably reflecting the involvement of processes along the STS (Scott & Johnsrude 2003). These results suggest differentiation in the anterior to posterior extent of the LSTG/MTG as a function of syntactic and semantic analysis processes (see also Caplan *et al*. 1996; Friederici *et al*. 2003; Hagoort 2003).

It is noteworthy that the areas implicated here in core language functions do not readily map onto the classical Broca and Wernicke regions (figure 1). The inferior frontal activations were not confined to Broca's area but generally extended beyond it to include BA 46 and 47. Similarly, the posterior temporal activation, which was strongest for syntactic analysis, was not confined to Wernicke's area. Indeed, most of the posterior temporal activity we observed was centred around the posterior MTG and IPL which border the Wernicke's area. This adds to the growing evidence that the regions comprising the neural language system are more extensive than originally thought (e.g. Dronkers *et al*. 2004) and include these more posterior temporal and parietal sites. Moreover, they also highlight that the LMTG, and not only the LSTG, is important in sentence-level processing. Although previous studies have reported activity for spoken sentences solely in the STG (Davis & Johnsrude 2003; Friederici *et al*. 2003), we consistently found maximal activity in MTG.

In summary, the studies we report here suggest that spoken language comprehension involves a network of posterior and frontal regions, with posterior regions being especially important in syntactic processing. These posterior areas include L posterior STG/MTG, angular gyrus, supramarginal gyrus and inferior parietal cortex, regions initially identified as having a significant role in language comprehension, by Marie & Foix (1917). The task for twenty-first century neuroscience is to use the imaging tools at our disposal in conjunction with well-developed cognitive models of language function to further elucidate the fine-grained structure of the neural language system.

## Figures and Tables

**Figure 1:**
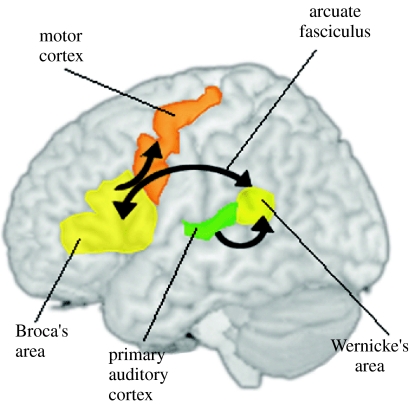
Classical model of the LH language system.

**Figure 2:**
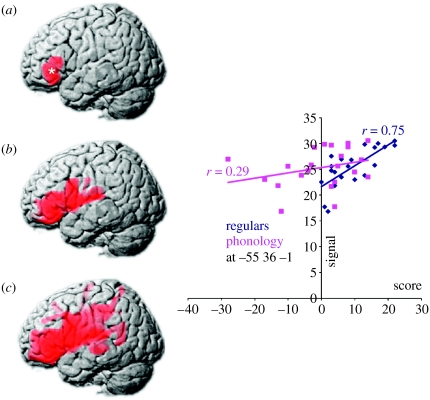
Structural correlates of regular inflection. Three-dimensional reconstructions of a T1-weighted MRI image showing brain areas where variations in signal density correlate with priming for regularly inflected words at: (*a*) *p*<0.001, (*b*) *p*<0.01 and (*c*) *p*<0.05 voxel thresholds. The clusters shown survived correction at *p*<0.05 cluster level adjusted for the entire brain. The statistical peak (−55, 36, −1) is in the left inferior frontal gyrus (BA 47), and the cluster extends superiorly into BA 45. At lower thresholds, the cluster extends from Broca's to Wernicke's areas and includes the arcuate fasciculus. The scatter plot shows the relationship between variations in signal density at the most significant voxel (see asterisk on (*a*)) and individual behavioural scores in the regular and the non-morphological phonological conditions. Adapted from Tyler *et al*. (2005*a*).

**Figure 3:**
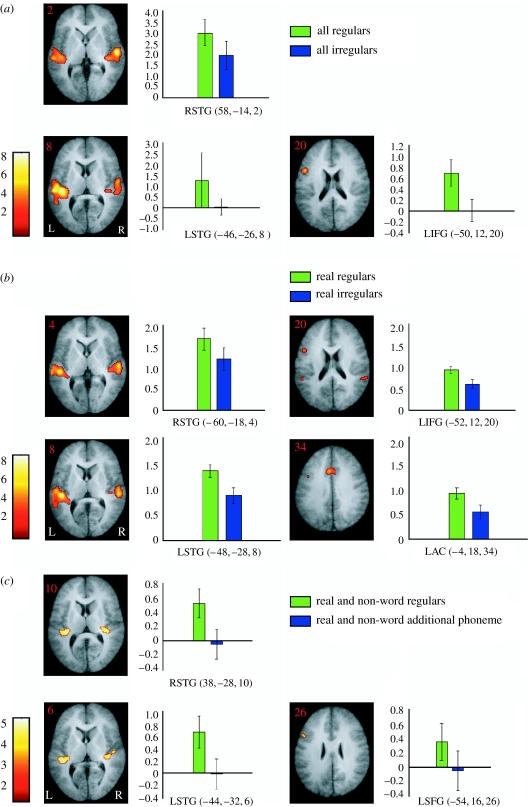
Functional correlates of regular inflection. The activations are superimposed on the mean T1 image of 18 volunteers. (*a*) Significant activations for the overall contrast of regulars (real, pseudo and non-word) minus irregulars (real, pseudo and non-word). Clusters were found in the RSTG, LSTG and LIFC. Activation peaks are given in brackets. (*b*) Significant activations for the contrast of real regulars minus real irregulars. Clusters were found in the RSTG, LSTG, LACC and LIFC. (*c*) Significant activations for the contrast of regulars (real and non-word) versus additional phoneme (real and non-word). Clusters were found in the RSTG, LSTG and LIFC. Adapted with permission from Tyler *et al*. (2005*c*). Copyright © Elsevier.

**Figure 4:**
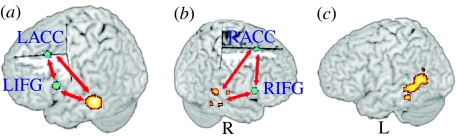
Functional connectivity analysis of regular inflection. Connectivity analysis in a group of healthy volunteers (20–40 years; based on data reported in Stamatakis *et al*. 2005). (*a*) The three-way interaction showing that the LACC predicts greater fronto-temporal interaction (LIFG and LMTG) in the context of regularly inflected when compared with irregularly inflected words. (*b*) The three-way interaction showing clusters in the RMTG that interact with activity in the RACC and RIFG in the context of regular versus irregular inflected forms. (*c*) The LMTG cluster predicted by the joint activity of RACC, RIFG for regular versus irregular inflected forms.

**Figure 5:**
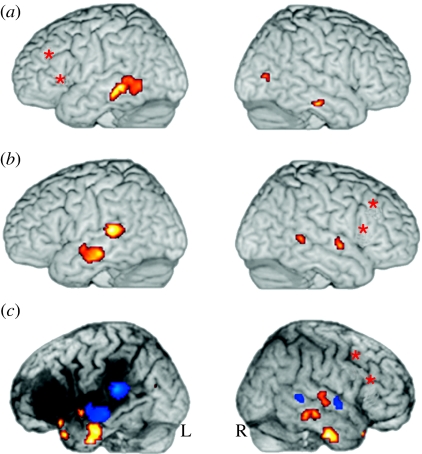
Functional connectivity for regular inflection following LH lesion. Connectivity analysis in (*a*,*b*) an age-matched control group and (*c*) patient P1, with extensive perisylvian damage. (*a*) The three-way interaction for a group of 40–60-year-olds between the two time series derived from the LH peak voxels in the subtractive analysis and the experimental condition (regulars versus irregulars). Predictor time series, derived from maxima in group activation patterns, are shown with asterisks in the LIFG and LACC. These regions predict activity in LMTG in the context of the experimental condition (regulars versus irregulars). (*b*) RH connectivity (for the contrast regulars–irregulars) for the 40–60 year-olds. Predictor time series are shown here with asterisks in the RIFG and RACC. These regions predict activity in LMTG as well as in the RH. (*c*) RH connectivity (i.e. three-way interaction between the two time series for the contrast between regulars and irregulars) for patient P1. Predictor time series, derived from maxima in P1's activation patterns (regulars–irregulars), are shown here with asterisks in the RIFG and RINS. The RH connectivity results from the controls (as in (*b*)) are shown in blue.

**Figure 6:**
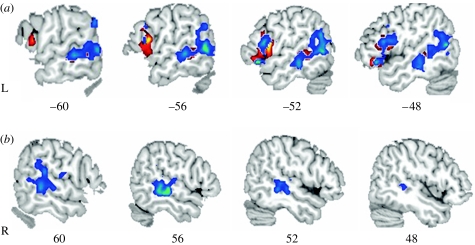
Contrasting effects of syntactic and semantic ambiguities. Significant activations (cluster threshold *p*<0.05 corrected for the entire brain, voxel threshold *p*<0.01 uncorrected) in LH and RH for (*a*) the contrast of semantically ambiguous–semantically unambiguous sentences (red) and (*b*) for the effect of syntactic dominance (blue; based on data reported in Rodd *et al*. 2004). The *x* coordinates are shown under each slice.

**Figure 7:**
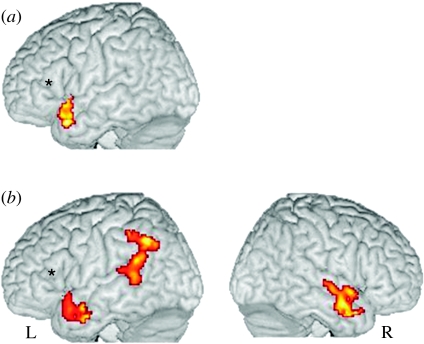
Functional connectivity analysis of syntactic and semantic ambiguity effects. Connectivity analysis using a predictor time series (marked by asterisks) found to be a statistical peak in the group (young normal) analysis. (*a*) The contrast of semantically ambiguous–unambiguous activity in the LIFG positively predicts activity in L anterior STG. (*b*) For syntactic dominance, activity in the LIFG positively predicts activity in bilateral anterior MTG/STG, L posterior MTG/STG and LIPL.

**Figure 8:**
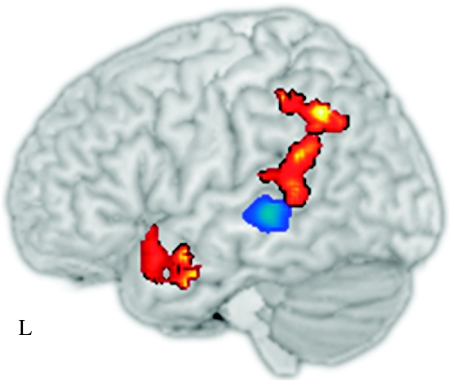
LH connectivity effects for regular inflection and for syntax. Results of connectivity analysis for syntactic dominance (red), from figure 7*b*, contrasted with parallel results for real regulars versus real irregulars (blue), from figure 4*a*, both for young controls (*p*=0.05). Predictor time series for both analyses were located in the LIFG.

**Figure 9:**
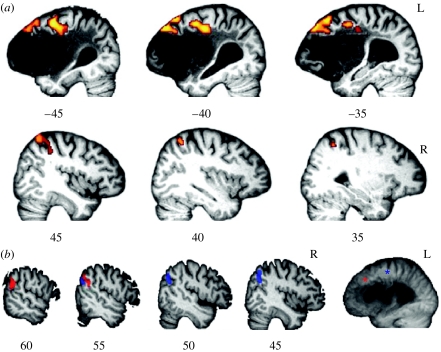
Syntactic ambiguity effects for patient P1. (*a*) LH and RH syntactic ambiguity activations, overlaid on sagittal slices of the patient's T1-weighted scan. The *x* coordinates are shown under each slice. (*b*) Connectivity analysis using predictors derived from P1's activation peaks (in L precentral G (blue asterisk) and LMFG (red asterisk)) for syntactic ambiguity, overlaid on the patient's RH. Activation in L precentral gyrus predicts activation in R angular gyrus (in blue); activation in LMFG predicts activation in R angular gyrus, extending to R supramarginal gyrus, RSTG and RIPL (in red).

**Figure 10:**
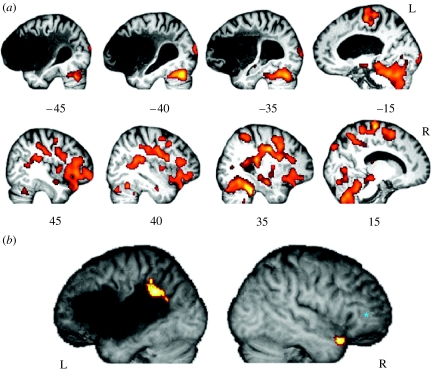
Semantic ambiguity effects for patient P1. (*a*) LH and RH semantic ambiguity activations for patient P1, overlaid on the patient's brain. The *x* coordinates are shown beneath each sagittal slice. (*b*) Connectivity analysis using predictors derived from P1's activation peak (see asterisk) for semantic ambiguity, overlaid on the patient's brain. Activation in RIFG, denoted by an asterisk, predicts activation in R anterior STG and L posterior MTG.

**Figure 11:**
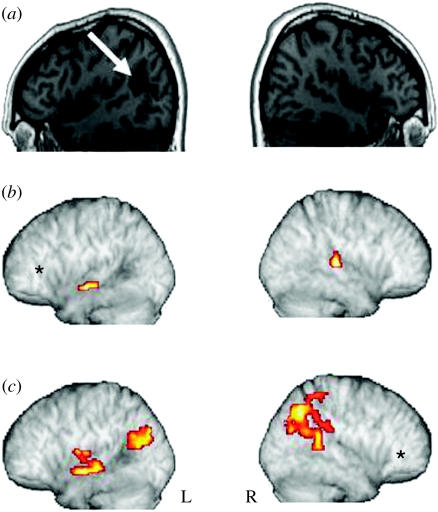
Semantic and syntactic connectivity effects for patient P2. (*a*) T1-weighted MR image for patient P2 (with lesion in L posterior MTG, indicated by a white arrow). (*b*) Connectivity analysis for semantically ambiguous words using predictors (see asterisk) derived from P2's activation peaks, overlaid on his three-dimensional reconstructed brain. Activity in the LIFG positively predicts activity in anterior regions of the LMTG and RSTG (BA 22, peak at MNI 62, −28, 4). (*c*) Connectivity analysis for syntactic dominance; activity in the RIFG, marked by an asterisk, positively predicts activity in anterior LMTG/STG and posteriorly in bilateral posterior MTG, and IPL.

**Figure 12:**
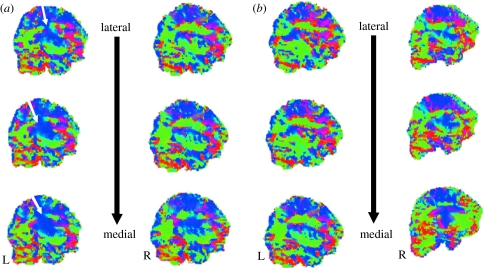
Disrupted white matter tracts in patient P2. Directional fractional anisotropy sagittal slices from (*a*) patient P2 (with L posterior temporal damage, see figure 11) and (*b*) an age-matched control. The colour maps are based on the principal diffusion directions: green, anterior to posterior; blue, inferior to superior; red, left to right. The arrows indicate a disruption in the LH arcuate fasciculus close to the patient's lesion.
